# Diversity of social-genetic relationships in the socially monogamous pied flycatcher (*Ficedula hypoleuca*) breeding in Western Siberia

**DOI:** 10.7717/peerj.6059

**Published:** 2018-12-06

**Authors:** Vladimir G. Grinkov, Andreas Bauer, Sergey I. Gashkov, Helmut Sternberg, Michael Wink

**Affiliations:** 1 Department of Biological Evolution, Faculty of Biology, Lomonosov Moscow State University, Moscow, Russian Federation; 2 Institute of Pharmacy and Molecular Biotechnology, Heidelberg University, Heidelberg, Germany; 3 Zoology Museum, Tomsk State University, Tomsk, Russian Federation; 4 OAG f. Populationsforschung Braunschweig, Braunschweig, Germany

**Keywords:** Extra-pair paternity, Microsatellites, DNA fingerprinting, Social systems, Monogamy, Polygamy, Pied flycatcher, EPP, Mating systems

## Abstract

We explored the genetic background of social interactions in two breeding metapopulations of the pied flycatcher (*Ficedula hypoleuca*) in Western Siberia. In 2005, we sampled blood from birds breeding in study areas located in the city of Tomsk and in a natural forest 13 km southward of Tomsk (Western Siberia, Russia). We sampled 30 males, 46 females, 268 nestlings (46 nests) in the urban settlement of pied flycatcher, and 232 males, 250 females, 1,485 nestlings (250 nests) in the woodland plot. DNA fingerprinting was carried out using eight microsatellite loci, which were amplified by two multiplex-PCRs and analyzed by capillary electrophoresis. About 50–58% of all couples were socially and genetically monogamous in both study plots. However, almost all possible social and genetic interactions were detected for non-monogamous couples: polygamy, polyandry, helping, adoption, and egg dumping. Differences in the rate of polygyny and the rate of extra-pair paternity between both study sites could be explained by differences in environmental heterogeneity and breeding density. Our findings suggest that egg dumping, adoption, polygamy, extra pair copulation, and other types of social-genetic interactions are modifications of the monogamous social system caused by patchy environment, breeding density, and birds’ breeding status.

## Introduction

Social monogamy is the predominant mating system of birds (Lack, 1968). However, the development of the DNA profiling method (Jeffreys, 1987) and its application in the study of birds’ mating systems has shown that social monogamy very often mixed with genetic polygamy and polyandry in nature. In socially monogamous bird species, extra-pair paternity (EPP) most often leads to a violation of genetic monogamy (Cleasby & Nakagawa, 2012; Griffith, Owens & Thuman, 2002). In general, birds with a low mortality, which can breed together in more than 1 year, have lower EPP rates than short-lived passerines that only meet for a single season (Wink & Dyrcz, 1999). EPP was detected in 86% of analyzed bird species (Griffith, Owens & Thuman, 2002). Furthermore, three-quarters of socially monogamous species (especially passerines) proved to be not completely genetically monogamous (Griffith, Owens & Thuman, 2002; Westneat & Stewart, 2003). Overall, true genetic monogamy was recorded in only 14% of surveyed passerine species, whereas genetic polyandry occurs regularly in the remaining 86% of species (Griffith, Owens & Thuman, 2002).

Variation of EPP rates were discovered between major avian lineages. The rates of individuals using alternative reproductive strategies (other than the EPP including intraspecific brood parasitism) are likely determined by fundamental interspecific differences in life history and parental care that evolved many millions of years ago (Arnold & Owens, 2002). Variation between populations or individuals of the same species, however, is more likely to be determined by differences in contemporary ecological and genetic factors. Several hypotheses have been postulated to explain EPP. Among adaptive scenarios, the “good genes” and “compatible genes” hypothesis are the most prominent and important of them (Griffith & Immler, 2009; Griffith, Owens & Thuman, 2002; Jennions & Petrie, 2000; Neff & Pitcher, 2005; for detailed review of possible benefits and costs associated with female extra-pair mating see Forstmeier et al., 2014). A meta-analysis study and researches on the lifetime reproductive success of extra-pair and within-pair young seriously questioned the applicability of these hypotheses to explain EPP (Akcay & Roughgarden, 2007; Hsu et al., 2014; Sardell et al., 2012; Schmoll et al., 2009; but see Gerlach et al., 2012; Griffith, 2007). Alternative hypotheses that consider female extra-pair mating to be a by-product of males’ coercion, genetic constraints as well as non-adaptive phenomena (intersexual antagonistic pleiotropy, pathological polyspermy, de novo mutations, or sexually transmitted diseases) are also posited (Forstmeier et al., 2014; Halliday & Arnold, 1987; Hsu et al., 2015; Moreno et al., 2015). Thereby the mechanism underlying female involvement in extra-pair mating remains unclear (Forstmeier et al., 2014; Griffith & Immler, 2009). The causes of incidence of such reproductive strategies and relationships in predominantly monogamous bird species as polygyny, polyandry, egg dumping, or helping are even less apparent (Alatalo et al., 1981; Alatalo & Lundberg, 1984; Dale et al., 1990; Huk & Winkel, 2006; Ilyina, 2012; Ratti, 1999; Schuett et al., 2017; Stenmark, Slagsvold & Lifjeld, 1988; von Haartman, 1969a; Yom-Tov, Wright & Both, 2000).

The pied flycatcher (*Ficedula hypoleuca*) is a very common and widespread Eurasian bird. Social monogamy is the main mating system in the species, which had been analyzed in several European populations with known demographic and phenotypic structure (Canal, Potti & Davila, 2011; Ellegren et al., 1995; Lehtonen, Primmer & Laaksonen, 2009; Lifjeld, Slagsvold & Lampe, 1991; Lubjuhn et al., 2000; Moreno et al., 2010, 2015; Ratti et al., 1995, 2001; Slagsvold et al., 2001; Tomotani et al., 2017). However, paternity analyses have not been performed for pied flycatchers nesting in the most eastern border of its distribution range—in Western Siberia. In this paper, we present results of a microsatellite analysis of two Siberian pied flycatcher metapopulations (urban and forest). We aimed to estimate the occurrence frequency of different types of social-genetic relationships in this metapopulations, and then we compared them and try to explain the observed differences. We also try to explain how revealed types of social-genetic relationships can appear in the pied flycatcher population.

## Material and Methods

### Field work

**Figure 1:**
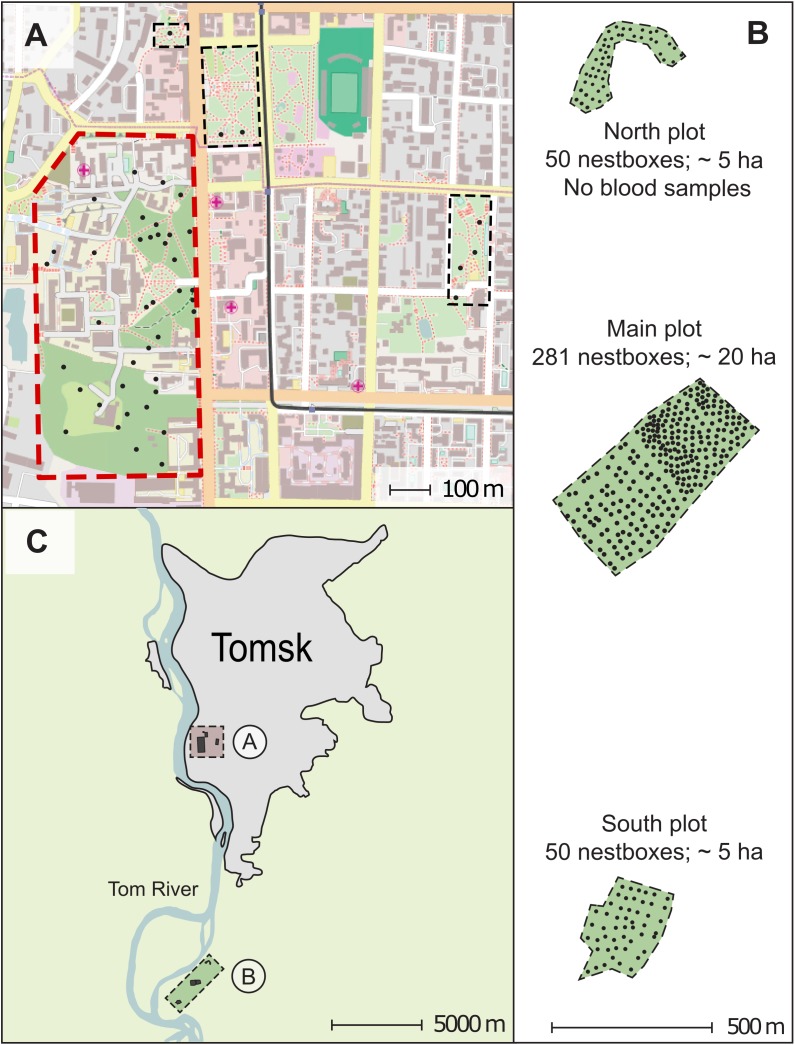
Map of the study area in Western Siberia, Russia. The figure parts show the urban plot map (A), the forest plot map (B), and the increased study area layout (C). Alphabetic labelling A and B on the figure part (C) correspond to the plot maps (A) and (B). The scales are shown for each map. Each small black circle indicates a nest-box. The polygon with a red solid dotted border indicates the area for which the nest-box density and the breeding density were calculated in the urban plot (A); the areas indicated by rectangles with a black thin dashed border were not used in these calculations. Green-filled polygons with a thin dashed border indicate areas for which the nest-box and breeding densities were calculated in the forest plot (B). The north-south orientation of the map in (B) is transformed to compact the figure. The city map data: © OpenStreetMap contributors.

The results presented in this paper were obtained during a long-term study of a pied flycatcher population in Western Siberia in the Tomsk region (Grinkov & Sternberg, 2018; Bushuev et al., 2012; Kerimov et al., 2014). This region constitutes the eastern boundary of the species distribution range. Investigations of pied flycatchers were launched in the Tomsk region in 1986 and continued until present (Kuranov, 2018). We used nest-boxes to attract these birds for breeding. We studied pied flycatchers on two main study sites—an urban and a forest plot. The nest-boxes in the urban plot were located directly in parks and boulevards of Tomsk. The main part of the nest-boxes area of the urban plot located within the Tomsk State University buildings complex (about 34 ha area) includes the territory of “University Grove” Park, the territory of “Reserve Park” of the Siberian Botanical Garden, as well as adjacent territories with planted trees between educational and economic buildings of the University (Fig. 1). Small-leaved deciduous tree species mostly constitute the tree layer of the nest-boxes area of the urban plot such as European white birch (*Betula pendula*), Black poplar (*Populus nigra*), Siberian linden (*Tilia sibirica*), Bird cherry (*Prunus padus*), Rowan (*Sorbus aucuparia*) with inclusions of coniferous trees such as Siberian fir (*Abies sibirica*), Siberian larch (*Larix sibirica*), Scots pine (*Pinus sylvestris*), Siberian cedar (*Pinus sibirica*). The maximum distance between most separated nest-boxes (maximum distance between nest-boxes) was 1.2 km.

The nest-box area of the natural settlement was established in 2001. It located 13 km southwards of Tomsk (56°21′N 84°56′E) in a mixed forest which consists of aspen (*Populus tremula*), birches (*Betula* spec.), spruce (*Picea abies*), Siberian fir (*A. sibirica*) and Scots pine (*P. sylvestris*). Aspen was the most abundant tree species in the stand, birches came in second place in view of their density. Other tree species were present as single isolated trees. The natural settlement of pied flycatchers was a forest area consisted of three subplots—two five ha and one 20 ha areas equipped with nest-boxes (Fig. 1). The distance between the most separated nest-boxes in the natural settlement was approximately three km. The urban and forest plots differed significantly in density of nest-boxes due to the differences in environmental structure (see below). The nest-box density in the urban plot has been 3.9 nest sites/ha, and the density of the nest-boxes in the forest plot ranged from 10 to 18.1 nest sites/ha depending on subplot (Fig. 1). In 2005, the actual breeding density of pied flycatchers was 1.32 pairs/ha in the city and 10.0 pairs/ha in the forest plot (specifically, 8.2–11.5 pairs/ha depending on subplots).

During breeding season, we regularly (once every 5 days) checked each nest-box to record the laying date of first egg, clutch size, brood size, and number of successfully fledged nestlings. We captured and ringed almost all females two times during breeding season. For the first time, each female was captured on day 7–9 of clutch incubation, and for the second time, a female was captured when feeding 9–11-day-old nestlings. At this time, a blood sample was collected from each female. Males were captured and blood-sampled when feeding 9–11-day-old nestlings. On the urban plot, plastic rings with a unique combination of colors were put on males and females in addition to the metal aluminum ring. It allowed remote identification of individuals without catching. Nestlings were blood sampled at day 10–12 after hatching. We also made a full series of morphological measurements (weight (by Pesola Microline spring balances 30 g, accuracy ±0.3%), wing and tail length (by metal ruler, accuracy ±0.5 mm), tarsus length (by ECOTONE Dial Callipers, accuracy ±0.1 mm), fat index, muscle score, moult score) each time when a pied flycatcher was captured.

We collected blood samples from birds breeding in both study plots in 2005. The pied flycatchers strongly prefer artificial nest sites (nest-boxes) over natural cavities. Therefore, we were able to trap almost all adults of the breeding flycatcher population. We sampled 30 males, 46 females, 268 nestlings (46 nests; 344 blood samples) in the urban settlement of pied flycatcher, and 232 males, 250 females, 1,485 nestlings (250 nests; 1,967 blood samples) in the forest plot. Blood samples in the forest plot were collected from pied flycatchers nesting in the subplot of 20 ha and in one subplot of five ha (Fig. 1). In the forest plot, we did not collect blood samples, only on the northern part (Fig. 1). In 2005, 41 nests of the pied flycatcher were found there, in which 41 females and 37 males were caught.

### DNA fingerprint analyses

All laboratory work was conducted at the Institute of Pharmacy and Molecular Biotechnology of Heidelberg University in Germany.

The blood samples were taken and stored afterwards in EDTA-buffer. After DNA extraction the pied flycatcher samples were analyzed using eight microsatellite loci FHU1/PTC2, FHU2/PTC3 (Ellegren, 1992); FHU3, FHU5 (Primmer, Moller & Ellegren, 1996); FHY336, FHY403, FHY427, FHY452 (Leder, Karaiskou & Primmer, 2008). The microsatellite loci were amplified using two multiplexes—PCRs (Set FHU, Set FHY). PCR was conducted as follows: 95 °C for 5 min, followed by 30 cycles 95 °C for 45 s, 56 °C for 1 min, 72 °C for 2 min, completed by a last step of 72 °C for 10 min. Amplified fragments were analyzed via capillary electrophoresis (MegaBACE 500 Sequencing System and MegaBACE 1000 Sequencing System; GE Healthcare Europe GmbH).

The degree of genetic relationships was determined by CERVUS 3.0 (Kalinowski, Taper & Marshall, 2007), a likelihood-based software. Characteristics of the molecular markers used in the study are presented in Table 1. To determine the paternity of each nestling the genotypes were compared with those of rearing parents in each nest. When the genotypes of the nestlings conformed to the genotypes of their social parents it was reasoned that social parents complied genetic parents. When there were observed mismatches in the genotype of a nestling in minimum two loci (or more) to its social father (the criterion “0 or 1 mismatch”), it was considered as a case of EPP. The analysis of paternity was conducted in CERVUS with all known males in a data set including the concerned nestling and the known genetic mother. CERVUS determines paternity using the highest LOD score (LOD: natural log of the likelihood ratio). The male with the highest LOD score was regarded as genetic father, but the LOD score was not the only criterion. Simultaneously the genotype of the suggested father was compared with the genotypes of the concerned nestling and the known genetic mother. Sometimes CERVUS suggested more than one male as possible genetic fathers, however, we did not observe such cases when there was more than one male that complied the criterion “0 or 1 mismatch.” Not in all cases CERVUS was able to find the genetic father in a data set. In cases of mismatches in a minimum of two loci the suggested father was discarded, and the concerned chick was treated as offspring of the known genetic mother and an unknown genetic father. A mismatching allele in a single locus was not regarded as EPP, but rather justified in mistakes in genotyping or caused by mutations (e.g., in primer binding site). In addition, in all these cases CERVUS was able to confirm the social father as the most likely genetic father.

**Table 1:** Characteristics of the molecular markers used in the study.

Locus	Expected heterozygosity	Observed heterozygosity, *1 − q*	Mean allele frequency, *q*	Probability of genotype sharing, *q*^2^(2 − *q*)	Probability of false inclusion, 2*q* − *q*^2^	Deviation from HW	Null allele frequency estimate
FHY 336	0.89	0.84	0.16	4.7 × 10^−2^	0.2944	Yes	1.73 × 10^−2^
FHY 427	0.85	0.79	0.21	7.9 × 10^−2^	0.3759	Yes	2.51 × 10^−2^
FHY 403	0.91	0.91	0.09	1.5 × 10^−2^	0.1719	Yes	3.10 × 10^−3^
FHY 452	0.85	0.84	0.16	4.7 × 10^−2^	0.2944	No	9.70 × 10^−3^
FHU 1	0.58	0.51	0.49	3.6 × 10^−1^	0.7399	Yes	7.29 × 10^−2^
FHU 2	0.75	0.72	0.28	1.3 × 10^−1^	0.4816	Yes	2.99 × 10^−2^
FHU 3	0.87	0.67	0.33	1.8 × 10^−1^	0.5511	Yes	1.33 × 10^−1^
FHU 5	0.92	0.84	0.16	4.7 × 10^−2^	0.2944	Yes	3.41 × 10^−2^

**Notes:**

Observed heterozygosity was calculated as (1 − *q*), where *q* is the mean allele frequency derived from CERVUS (following Kalinowski, Taper & Marshall, 2007). For a single locus the probability that two unrelated individuals will share the same genotype is given by *q*^2^(2 − *q*) (Kalinowski, Taper & Marshall, 2007), where *q* is the mean allele frequency (following Kalinowski, Taper & Marshall, 2007). For a single locus the probability of false paternal inclusion is given as 2*q* − *q*^2^ (Kalinowski, Taper & Marshall, 2007); Null allele frequency estimate: calculated in CERVUS.

HW, Hardy–Weinberg equilibrium.

### Estimation of environmental heterogeneity

We assessed the environmental heterogeneity qualitatively in terms of the presence/absence of buildings, roads, glades, lawns, open areas without trees (Fig. 1). Besides, the urban plot had a patchy distribution of forest areas suitable for placing nest-boxes. Because of this, the studied area with nest-boxes in the city was highly fragmented, nest-boxes were patchy distributed, and divided by areas without trees unsuitable for placing nest-boxes. Blocks of flats and office buildings also created additional visual and acoustic isolation between nest-boxes and their owners (Fig. 1). There was not a single sign of heterogeneity from the above in the forest plot. Moreover, the forest making up of the nest-box area is very homogeneous in terms of tree species. Thus, we estimated the differences between plots in terms of structural environmental heterogeneity, rather than the variety of species composition and species richness in these biotopes.

### The definitions used in the paper

Social monogamy or monogamy refers to a male and female’s social living arrangements (e.g., shared parental care) without inferring any sexual interactions or reproductive patterns (Reichard & Boesch, 2003). Genetic monogamy is a condition in which the paternity analysis has shown that all the nestlings in the brood of the pair hatched from eggs laid by the within-pair female and sired by the within-pair male. EPP is defined as fertilization resulting from copulations (extra-pair copulations, EPC) outside social bonds (Davies, 1991; Møller, 1986; Westneat, Sherman & Morton, 1990). In socially monogamous species, extra-pair young (EPY) are those sired by males other than the putative social father (Owens & Hartley, 1998).

It is known that not all socially polygynous males feed their chicks in all broods (Huk & Winkel, 2006; Lundberg & Alatalo, 1992; von Haartman, 1969b). Therefore, we used two methods to define social polygyny. The male acquired the status of polygynous if it was caught and/or was seen (only in the urban plot) while feeding nestlings in two or more nests (the primary method) and if paternity for two or more complete broods was confirmed for him by genetic markers (an additional method). In the forest plot, only nine polygynous males were found. Four of them were first identified by catching, and later their status was confirmed by the paternity analysis. Five males were identified by the paternity analysis. Two of these five males were assigned to broods where we could not find males that assisted the females to feed the nestlings. Next two of these five males were assigned to broods were other males were captured. Finally, one of these five males, a trigynous male, was caught on nestlings in one nest, then his paternity was confirmed for two more nests. In one nest there was a female without a male assistance, and in another nest another male was caught. In the urban plot, 10 polygynous males were found. Nine of these males were originally identified as polygynous by catching and visual observations with subsequent confirmation by paternity analysis. One male was assigned to brood by paternity analysis where we could not find a male that assisted the female to feed the nestlings.

We applied the term “foster parents” to those parents who fed non-descendant nestlings resulting from egg dumping and joint clutches. “Helper” status has been assigned to individuals who fed the genetically non-related nestlings together with the genetic parents (Ilyina, 2012). We used the term “adoptive parents” in cases where all the nestlings were not related to rearing birds (Ligon, Liu & Hill, 2009; Morris, Woulfe & Wichert, 1991; Riedman, 1982). All these types of parental care can be designated by the term alloparental care, which was defined as any form of parental care directed towards non-descendant young (Wilson, 1975).

**Figure 2:**
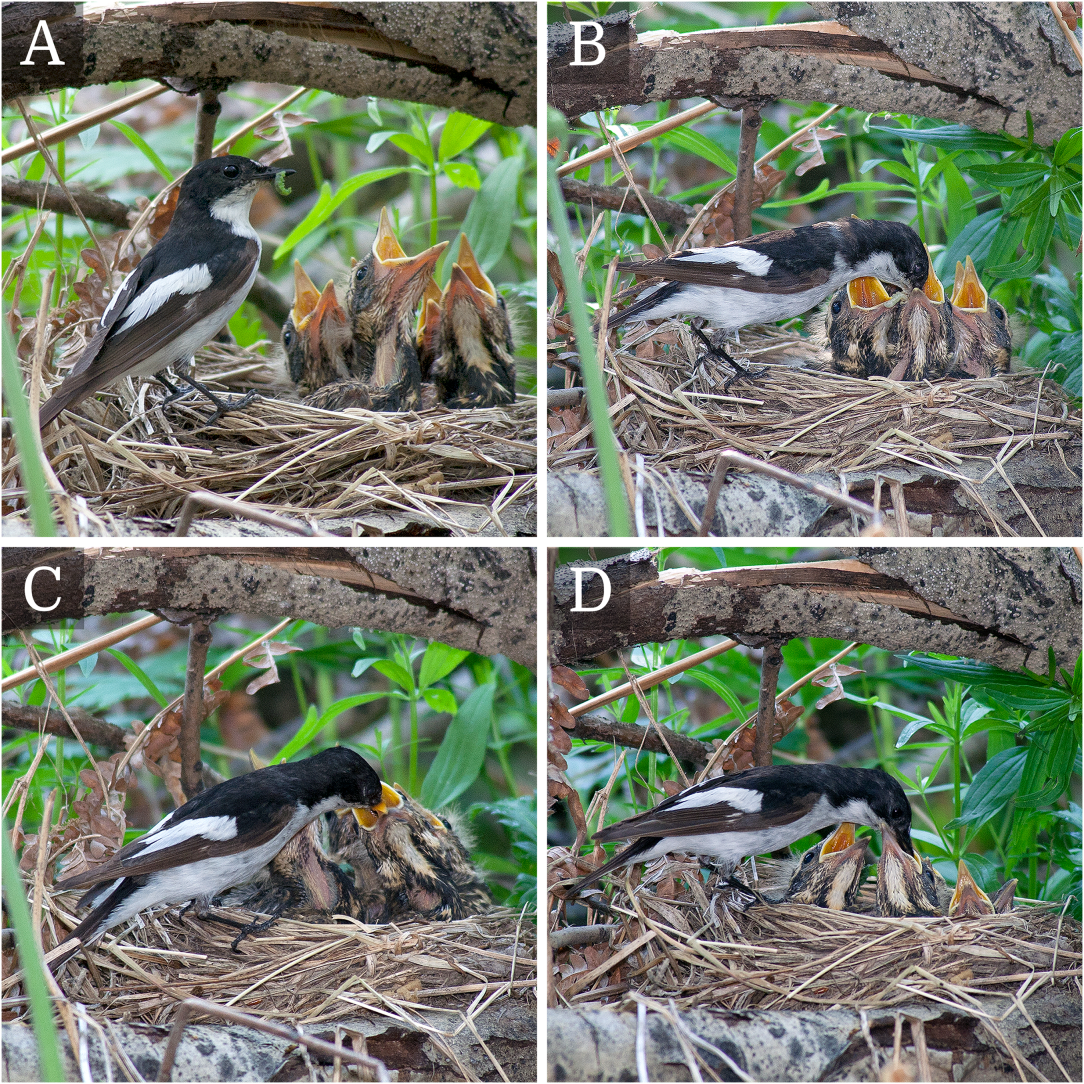
The male of the pied flycatcher (*Ficedula hypoleuca*) feeds the chicks of the redwings (*Turdus iliacus*) despite the fact that he has his own nestlings in the nearby nest-box. The figure parts (A–D) are photos of different visits of the pied flycatcher male with food to the chicks of the redwings and their subsequent feeding. Photo credit: Vladimir G. Grinkov.

We believe that the sexual and social behavior (e.g., sexual monogamy, EPC, polygamy, adoption) of the pied flycatcher can be inferred or deduced from recording the feeding behavior and analyzing paternity with a high degree of certainty, but not in all cases. The fact is that among the pied flycatcher, there are relatively rare cases where not only parents feed nestlings, but also not related birds (non-breeders, birds that have lost their brood, neighbors) (Ilyina, 2012; Kallander & Smith, 1988; our experience; Fig. 2). That is why there may be cases when it is difficult to determine the social status of the individual. For example, if a bird completely lost paternity or completely gained paternity because of the EPC, then the status of such birds cannot be accurately determined using only catching and paternity analysis without EPC registration. A completely lost paternity male becomes indistinguishable from an alloparent, because it is impossible to affirm whether this male has been mating with a social female. The male, who gained full paternity because of the EPC, becomes indistinguishable from a polygynous male. If the male lost his paternity in his nest and got it in another, and then this male was caught in another nest during the feeding of the young (Ilyina, 2012; Kallander & Smith, 1988; Ottosson, Backman & Smith, 2001; Schuett et al., 2017), then such a male is indistinguishable from a monogamous male. In all such rare cases we have applied the principle of parsimony: if there are several logically consistent explanations of a phenomenon explaining it equally well, then, other aspects being equal, the simplest of them should be considered true (Sober, 2015).

Therefore, if in a particular nest all nestlings genetically belonged to a non-rearing male, which already had another nest in the study area, we changed the status of rearing male from “father” to “helper,” and the previous status of a non-rearing male to “socially polygynous male” (see above definition for polygynous males). Corresponding changes we have made for the female status. If all nestlings genetically belonged to a non-rearing male which was not captured and blood sampled (unknown male), we changed the status of rearing male to “helper,” and the status of rearing female to monogamous, whose initial social partner has disappeared (e.g., due to predation). If all nestlings genetically belonged to more than one extra-pair male, we changed the status of rearing male to “helper,” and the status of rearing female from monogamous to “individual involved in EPC.” Accordingly, to separate cases of EPC from others we need to use such formal criteria: (1) all chicks in the brood were genetically direct descendants of a rearing female; (2) in a nest there are nestlings sired at least by two males; (3) and finally, at least one nestling was genetically a direct descendant of a rearing male.

Thus, all the terms we use are conditional and the groups of birds they designate are heterogeneous. For example, “helpers” can include both real helpers and males who have completely lost their paternity (however, the probability of such an event is very low, please see the results). Monogamous males can include real monogamous males, extra-pair males gaining full paternity, and polygynous males having additional nests outside the study area. Polygynous males can include both polygynous and extra-pair males gaining full paternity because of the EPCs (but such an event has the lowest likelihood, please see results).

### Ethics statement

Our work conforms to the legal requirements of the Russian Federation as well as to international ethical standards. All our treatments and samplings have been intravital and have not required prolonged treatment and handling of birds. The species from our study is not included in the “Threatened” category of the IUCN Red List of Threatened Species. Bioethics Commission of Lomonosov Moscow State University has provided full approval for this research (Protocol No 89-o of March 22, 2018).

## Results

In the following, we have analyzed our genotyping data from the perspective of individual broods and the corresponding parents but also from individual nestlings. From “nestling point of view” there are only four possible situations: (1) genetic parents are also the social parents; (2) an alloparental male and a genetic mother feed a nestling; (3) a genetic father and an alloparental female feed a nestling, and finally; (4) both alloparental male and female feed a nestling.

### Monogamy

We found statistically non-significant differences in the proportion of monogamous couples between both study plots (Chi-squared test, *p* > 0.05): in the forest plot genetic monogamy accounted for 58.0% of couples (145 out of 250 broods), whereas it was 50.0% (23 out of 46) in the city plot (Table 2). In 42.0% (forest plot) and 50.0% (city plot) of the families, we found quite a diversity of social and genetic relationships.

**Table 2:** Social and genetic relationships in the pied flycatcher breeding in Western Siberia.

Plots	Relationships	*N* nests	In combination with						
			Female replacement	Male replacement	Parent replacement	Egg dumping	Polyandry	EPC in males	EPC in females
Forest	Monogamy	145	8	4	4	6	–	–	–
	EPC in males	29	3	–	–	2	–	29	–
	EPC in females	52	–	1	–	3	1	8	52
	Polygamy	16	–	2	–	1	–	2	–
	Trigamy	3	–	1	–	–	–	1	1
	Polyandry	5	–	–	–	1	5	–	–
	Total	250	11	8	4	13	6	40	53
City	Monogamy	23	–	–	–	1	–	–	–
	EPC in males	1	–	–	–	–	–	1	–
	EPC in females	2	–	–	–	–	–	–	2
	Polygamy	20	1	–	–	–	–	2	1
	Total	46	1	–	–	1	–	3	3

In general, from “nestling point of view,” of 1,485 young produced in the forest plot, 1,217 (81.9%) nestlings were reared by genetic parents, 142 (9.6%) nestlings were reared by genetic mothers and alloparental males (genetically unrelated birds), 93 (6.3%) nestlings were reared by genetic fathers and alloparental females, and finally 33 (2.2%) nestlings were reared by alloparents.

In the urban plot, of 268 nestlings, genetic parents cared for 255 (95.15%) chicks; genetic mother and alloparental males reared 4 (1.49%) chicks; genetic fathers and alloparental females reared 8 (2.99%) chicks; alloparents reared 1 (0.37%) chick. These differences between sites were statistically significant (Chi-squared test = 32.1, d*f* = 3, *p* < 0.01).

### Extra pair paternity

In the forest plot, we discovered 53 broods (21.2%) with more or less clear evidence that females took part in EPCs and produced EPY (Table 3). Among the promiscuous females, 45 females (84.9% of the promiscuous females or 18.0% of all breeding females) produced young with two males (within-pair male plus extra-pair male), seven females (13.2% of the promiscuous females or 2.8% of all breeding females) with three males and one female (1.9% of the promiscuous females or 0.4% of all breeding females) with four males. From the above calculations, it can be concluded that the pied flycatcher females can copulate with more than one male, but the proportion of such females is very small.

**Table 3:** The proportion of EPY in the pied flycatcher broods in the forest and the urban plots.

Plots	*N* of nests	*N* of EPMs	*N* of EPY	*N* of nestlings in a nest	EPY proportion
Forest	26	1	1	3–8	0.13–0.33
	13	1	2	3–8	0.25–0.67
	1	1	3	7	0.43
	3	1	4	5–6	0.67–0.80
	2	1	5	7–8	0.63–0.71
	4	2	2	5–6	0.33–0.40
	1	2	5	5	1.00
	1	2	4	6	0.67
	1	2	6	6	1.00
	1	3	3	6	0.50
City	2	1	1	5–6	0.17–00.20
	1	1	2	7	0.29

We also found, that 40 known males (17.2%) had EPP. Among them, 34 males (85.0%) had young with two females, five males (12.5%) with three females, and finally, one male (2.5%) with four females. DNA data revealed that eight females (20.0%) of those males, which participated in EPC, in turn also copulated with extra-pair males.

In the urban plot (Table 3), we discovered three cases of EPP. Only three females (6.5%) had EPY. All these females had two sexual partners. We found three males (10.0%) which sired nestlings outside their social bonds. One of these males also had only two sexual partners, and another two males were bigamous and copulated with three females.

Overall, the rate of EPP among males in the forest plot has been 1.7 times higher than the one in the urban plot (statistically insignificant, Chi-squared test, *p* > 0.05); and the rate of EPP in females in the forest plot has been 3.3 times higher than the one in the urban plot (Chi-squared test = 6.3, d*f* = 1, *p* < 0.05).

It should be noted that the nests in which we revealed many EPY (four to five) are rare. In two cases where there is a proportion of EPY equal to one (Table 3), females mated with more than one male (Table 3). Thus, the probability of total loss of paternity (for a whole brood) due to EPC is extremely low and is possible only when females copulated with many extra-pair males. Consequently, the probability of gain of total paternity (for a whole brood) for one male due to EPC is also extremely low.

### Polygyny

It has not always been easy to identify polygyny in our material (see Methods). Formally, in the forest plot we found 19 broods out of 250 (7.6%) that were sired by eight bygynous and one trigynous males (3.9% of all breeding males). Remarkably, we could not find evidence that the females of the bygynous males produced EPY. One female of the trigynous male had two EPY. In turn, three polygynous males (33.3%) copulated with extra-pair females. In the urban plot, 20 broods (43.5%) with polygynous males were detected. One female of the polygynous males had extra-pair nestlings, and two polygynous males (20.0%) produced EPY with extra-pair mates. Thus, in the urban plot 10 out of 30 captured males (33.3%) had two females.

Therefore, the rate of polygyny among males in the urban plot has been 8.5 times higher than in the forest plot (Chi-squared test = 12.2, d*f* = 1, *p* < 0.001).

### Bigyny with a single brood and polyandry

In six broods the corresponding male sired young with two females, one of which feeds the nestlings (Table 4). In two cases, we could identify the second female. These two females had their own nests not far away. These females behave like polyandry birds (Tables 2 and 4). As far as we know this is the first time that polyandry has been discovered in the pied flycatcher. In the urban plot, there were no such cases.

**Table 4:** Evidence for polyandry in the pied flycatcher in the forest plot, and the proportion of nestlings from an extra-pair female.

*N* of nests	*N* of nestlings in a nest	*N* of nestlings from an extra-pair female	Proportion	Notes
1	6	1	0.17	Polyandry in female? Polygynous male or EPC male? In each nests, we have identified all mothers and father for all nestlings
1	6	2	0.33	
1	7	1	0.14	Polyandry in female? Polygynous male or EPC male? In each nests, we know father for all nestlings, and we know at least one mother, some fledglings from unknown female
1	6	1	0.17	
1	7	2	0.29	
1	7	3	0.43	

### Egg dumping

Egg dumping or intraspecific brood parasitism occurs when a female lays her egg(s) in the nest of another bird. Egg dumping is the third most common phenomenon in the population (Tables 2 and 5). We have decided that a chick comes from a “dumped” egg, when its genotype does not match those of its parents. In the forest plot, we found 13 nests out of 250 (5.2%) in which the genotype of a nestling has not been related to the corresponding parents. Of 1,485 genotyped young flycatchers, 13 (0.88%) were considered as evidence for egg dumping. The number of such genetically non-related nestlings varied from one to four in a clutch. A total of 61.5% of the broods with egg dumping contained a single dumped egg only (Table 5).

**Table 5:** The proportion of egg dumping in the pied flycatcher in the forest plot.

*N* of nests	*N* of nestlings in a nest	No of “dumped” nestlings	Proportion	Notes
1	7	1	0.14	Minimum one fledglings genetically related to rearing adults, we do not know mother and father of “dumped” nestlings
5	6	1	0.17	
1	5	1	0.20	
1	4	1	0.25	
2	7	2	0.50	
2	6	3	0.5	
1	5	4	0.80	

In the urban plot, only one case of egg dumping (one nestling out of 268 genotyped young flycatchers; 0.4%) was detected.

### Female, male, and parent replacement

We also found genetic evidence for female, male, and parent replacement (Tables 2 and 6). In the forest plot we discovered 11 broods (4.4%) which were social genetically not related to females attending the nest but were sired by the male attending the nestlings. In eight broods we found evidence for the replacement of the corresponding male (3.2% of all broods). In four broods (1.6%) the young were not related to neither their social father nor mother.

**Table 6:** Evidence for replacement of mates in the pied flycatcher in the forest plot.

Type of replacement	*N* of nests	*N* of nestlings in a nest	*N* of genetically unrelated nestlings	Notes
Female replacement	3	5	5	All nestlings are not genetically related to within-pair female
	6	6	6	
	1	7	7	
	1	8	8	
Male replacement	1	1	1	Nests of bigynous males? All nestlings are not genetically related to within-pair male, but sired by known extra-pair males
	1	6	6	
	1	7	7	
	1	6	6	All nestlings are not genetically related to within-pair male, and sired by two unknown extra-pair males
	1	6	6	All nestlings are not genetically related to within-pair males
	1	5	5	
	1	7	7	
	1	8	8	
Parent replacement	2	5	5	All nestlings are not genetically related to reared adults
	2	6	6	

In the urban plot, we found one case of female replacement.

## Discussion

The main aim of this study was a general assessment of the pattern of social-genetic interactions in Siberian pied flycatchers. Our study revealed a high degree of monogamy but also a wide variety of other social-genetic relationships in the population (Table 2). We also discovered that even in a single nest, nestlings of different genetic origin might be present (Table 2), and that some birds can copulate with up to four partners. The proportion of non-descendant young in the brood was shown to vary from 13% to 100% (Table 3). In contrast to earlier published data (Lubjuhn et al., 2000), our study revealed, that broods of polygamous males show a lower rate of EPY than those broods of socially monogamous individuals. Polygynous males, as also shown in our study, could participate in EPC and gained additional paternity along with socially monogamous males. One of the most unusual and unexpected results was the detection of polyandry in females, which is not a result of EPC. We were not able to find reports of similar cases for the pied flycatcher in the literature.

The second important goal of the work was to explain how revealed types of social-genetic relationships can appear in the pied flycatcher population. In the following discussion sections, we outline our interpretation.

### Alloparental care due to egg dumping, adult replacement, adoption, helping, and polyandry

Most cases of non-monogamous social-genetic interactions in the pied flycatcher can probably be explained by the reproductive state of a bird. For example, we think that egg dumping happens in most cases as the result of the requirements to finish the launched process of egg formation. It takes 4 days in the pied flycatcher to produce one egg (Romanoff & Romanoff, 1949; King, 1973 as cited in Ojanen, 1983). Consequently, if a female laid its first egg, there are four other eggs in her body in different stages of development and these eggs must also be laid. If a female lost her nest or nest hole due to predation or other reasons, such a female will try to lay the rest of the clutch into other nests, in most cases, of the same species of birds (Holmes, 1990; von Haartman, 1969b), but sometimes even in nests of other species (Jarvinen, 1993). Sometimes, a female laid the complete rest of the clutch into a single nest and started to incubate the new joint clutch together with former owners of the nest, because the latter birds were tolerant to the presence of the bird who had lost the nest. In such cases, we often find an exceptionally large clutch size (11–14 eggs).

Sometimes, we were able to catch two females in a particular nest-box, where they both incubated eggs and fed nestlings. This might be interpreted as if a male had “attracted” two females. Some researchers described such a situation as “monoterritorial bigyny” (Winkel, 1998). We assume, however, that the situation has not been the result of true polygyny. This is the result of twin (combined) clutches, when a pair of birds tolerates the female that joined them to finish the clutch. If the first female-owner disappeared (due to predation), we could find the case of “female replacement” or “adoption.”

Male, female, and adult replacement may also happen as the result of an instinctive urge to feed nestlings. If a bird lost its own nestlings due to predation, for example, such a bird can join another couple and start to “help” them feed their nestlings. If we catch such a bird or birds instead of the first owners of the nest-box, and based on genetic control, we might interpret them as male, female, or parent replacement (adoption).

Some birds are so overstimulated that they can start to “help” feeding nestlings from birds of other species, and at the same time continue to feed their own nestlings. As documented in Fig. 2, a male pied flycatcher feeds the nestlings of the redwing (*Turdus iliacus*) simultaneously to its own chicks. The literature also describes the case in which a pair of the pied flycatcher fed simultaneously fieldfare (*Turdus pilaris*) and their own chicks (Zubkova, 2013). Moreover, it has been shown, that the pied flycatcher adults fed fieldfare chicks more often than their own chicks. The pied flycatcher continued to feed its own chicks in a normal rate after the fieldfare chicks had fledged (Zubkova, 2013).

The occurrence of alloparental care in the studied population may seem too high. However, even though the pied flycatcher is one of the most studied species of birds, many aspects of its biology are still poorly understood. For example, the behavior of non-breeding birds has not been studied in practice. The proportion of such individuals appeared to be colossal. According to some estimates, non-breeders comprise 60–80% of the first-year-old birds (Sternberg, 1989), and the population reserve (the number of individuals not participating in the reproduction) can many times exceed the reproductive part of the population (Grinkov & Sternberg, 2018; Both et al., 2017; Sternberg et al., 2002). An alloparental care of non-breeders and birds who have lost broods appears to be more common than previously suspected (Ilyina, 2012; Kallander & Smith, 1988). Against this background, and considering the very high breeding density of the Tomsk population in the forest plot, these figures do not look very large. Yet in very deed the number of such cases can be overestimated, because we can occasionally catch genetically non-related birds feeding nestlings, for example, neighbors (Ilyina, 2012; Ottosson, Backman & Smith, 2001; Schuett et al., 2017). However, it is also true that alloparents are existent in this species. Estimating more precisely the proportion of such birds is possible only in studies in which the behavior of specimen is recorded in every detail.

### Extra-pair copulations, polygyny

Polygyny and EPC seem to be very complex events among birds in general, and in the pied flycatcher in particular; they are not static but plastic traits (please see the introduction, which sets out the main views on the mechanisms of their evolution). In general, mating systems, even if the mechanisms of their evolution are unknown, can be influenced by environmental heterogeneity, asynchrony in sexual receptivity and operational sex ratio (OSR) (Emlen & Oring, 1977; Westneat, Sherman & Morton, 1990; Wittenberger, 1979). Patchy distributions of critical resources could lead to a skewed distribution of females, and asynchrony could allow males to attract and to form a new pair after courting and copulating with the first mate (Emlen & Oring, 1977; Westneat, Sherman & Morton, 1990; Wittenberger, 1979). Polygyny can be expected, when the ratio of the number of unpaired females available to an average male exceeds one (Emlen & Oring, 1977). Nevertheless, even when there are unpaired females, males may still have opportunities to fertilize paired females. Therefore, OSR can be divided into two components: (1) the ratio of sexually active males to unpaired females (potential for polygyny), and (2) the ratio of the number of sexually active males to fertilizable, but paired, females (potential for EPC) (Westneat, Sherman & Morton, 1990). Westneat, Sherman & Morton (1990) suggested that environmental homogeneity should greatly reduce the opportunities for males to attract unpaired females, and homogeneity will affect the potential for EPC far less than the potential for polygyny. Additionally, it may be a time allocation trade-off in gaining polygynous or EPC status for a male. Obtaining exclusive sexual access to secondary mates requires territorial defence and self-advertising, whereas leaving the territory unguarded and seeking out receptive females is necessary to gain EPC status (Westneat, Sherman & Morton, 1990). Therefore, these behavioral patterns of birds that increase incidence of polygyny will potentially decrease the pursuit of EPC (Westneat, Sherman & Morton, 1990).

Our results seem to be in a good agreement with these assumptions. Higher incidence of polygyny (about 8.5 times more) and lower of EPP (about 3.3 times lower for females and 1.7 times lower for males) are typical for the urban plot in comparison to the forest plot. The most important differences between these plots were the nest-boxes density and the environmental patchiness. Wide roads, squares, large areas without trees and buildings made the urban environment extremely patchy, and the visual and acoustic communication between birds was more restricted. We assume, that in spring it is difficult for male flycatchers to find nest sites in the urban plot, and we hypothesize that there is a male shortage in the urban plot. Under such conditions, males had higher chances to defend several territories (nest-boxes) within a small area, long enough to attract a second mate; and at the same time these males had more difficulties to pursue EPC (as the next female has been far away or less detectable). It was demonstrated that EPC occurs mostly between closely residing birds (Canal, Jovani & Potti, 2012). In the more homogenous environment of the forest plot with high nest-box density, males were in an opposite situation. The very high breeding density increased the chance of EPC for males. Therefore, the differences in the rate of polygyny and the rate of EPP between the urban and the forest plot can be explained by their differences in environmental patchiness and breeding density.

### Social monogamy as a capacitor for mating and social behavior evolution

Herewith, we argued that social monogamy can generate, as a by-product, all types of social-genetic relationships resulting in the alloparental care (egg dumping, adult replacement, adoption, and helping). Recent studies have shown that the EPC among the pied flycatcher can be the result of sexual coercion, and is mainly driven by extra-pair male pursuit, capable of overruling female avoidance and mate defence by within-pair males (Moreno et al., 2015). Therefore, on the individual level, EPP can occur as a side-effect of a bird’s breeding state, because males in the period of fertility make attempts to mate with all females who may be encountered in the nearest environment. Polygyny can also be a kind of side-effect of monogamy. Polygyny seems to be the consequence of a male’s polyterritoriality (Lundberg & Alatalo, 1992; Silverin, 1975; von Haartman, 1956, 1969a), which in turn depends on its physiology. For example, the polygynous status can be induced by hormonal stimulation of males (Silverin, 1980; Silverin & Wingfield, 1982; Wingfield, 1984). Subcutaneous implants of testosterone into males of monogamous species enhance frequencies of territorial aggression and courtship behavior, resulting in a marked increase in the size of the territory (Wingfield, 1984). A substantial number of such testosterone-implanted males of monogamous species also becomes polygynous, attracting two and sometimes three females to settle on their enlarged territories (Wingfield, 1984). In our experience, almost all pied flycatcher males are potentially polyterritorial in early spring: if a male has a chance, it will advertise more than one nest-box in the study area. Arrival of new males during migration will gradually restrict polyterritoriality thus decreasing the chance to attract a secondary mate.

As far as we know, no evidence exists that polygyny and other types of non-monogamous mating in the pied flycatcher is a heritable or a repeatable trait. The mating status of a male is determined in each breeding season de novo, and this status depends on very specific circumstances such as environmental heterogeneity, breeding density, the breeding condition’s level of development or shortage of partners. Among males the mating status is part of a mixed reproductive strategy rather than a specialized reproductive behavior adopted exclusively by a subset of the population (Beecher & Beecher, 1979; Fitch & Shugart, 1984; Trivers, 1972; Westneat, Sherman & Morton, 1990).

Therefore, we assume that egg dumping, adoption, polygamy, or EPC are modifications of monogamy which occur in very special situations, including an increased breeding density in a relatively homogenous environment. It is highly likely that selective mechanisms are not needed for development of all types of non-monogamous relationships in a population of the monogamous bird species such as the pied flycatcher (Hsu et al., 2015). All non-monogamous relationships arise within the reaction norm of monogamous behavior in response to changes in environmental conditions (environmental patchiness, breeding density, predator pressure, surplus, or shortage of mating partners). In its broadest sense, our results may be an example of how novel features may evolve in natural populations (in this case, new types of mating and social behavior). Novelties often arise suddenly in response to unusual conditions and are not controlled directly by natural (Darwinian) selection (Moczek, 2012).

## Conclusions

The main challenge of this study was to obtain a complete set of samples from all nesting birds in the large study area for subsequent analysis of their genetic bonds. As far as we know, such extensive studies, in which 296 broods were examined for one reproductive season, have not yet been carried out on the pied flycatcher. Studies based on a dataset comparable to our sample size were conducted on some European populations (Canal, Jovani & Potti, 2012), but these materials were collected during several breeding seasons of the species. Analysis based on an extensive sampling revealed very complex social-genetic relationships of birds, which can vary depending on environmental conditions (environmental heterogeneity, breeding density). It allowed us to assume that social monogamy can produce all other relationships as a side effect.

However, during our work we faced great difficulties. For example, in case of broods with a high proportion of a non-descendant young number, problems occurred classifying the different types of genetic and social relationships between individuals using the most common definitions of such relationships. All these difficulties are indicated in Tables 4–6 in the column “Notes.” This is partly because we could not record the behavior of all pied flycatchers in the wild. This lack of information of the real behavior of birds in nature makes it difficult to systematize different types of social-genetic relationships, based solely on genetic data (e.g., see “The definitions used in the paper” in the Materials and Methods). It appears easier to discuss this issue based on the paternity of individuals and not that within broods (the classification from “the nestling point of view,” see Results). This classification is simpler, since it does not depend on the definitions, on whether we know the actual behavior of birds in nature, and on the incompleteness of control of the studied population. We believe that such a classification is much better suited for comparing different populations by the rate of true monogamy and other types of social-genetic relationships.

The work was conducted only during one reproductive season in the Western Siberian population of the pied flycatcher, but in areas that differ greatly in the breeding density of birds. Accordingly, we were able to obtain only spatial patterns in the variation of social and genetic relationships between birds in the study areas. However, we believe that this is sufficient for a basic description of the diversity of social-genetic links between individuals of the studied population. Subsequent studies in this region are likely to reveal the temporal pattern of the variations in the proportion of monogamy, EPP, and other types of social-genetic relationships between birds.

## Supplemental Information

10.7717/peerj.6059/supp-1 Supplemental Information 1 Raw data: Diversity of social-genetic relationships in the socially monogamous pied flycatcher (Ficedula hypoleuca) breeding in Western Siberia.

## Additional Information and Declarations

### Data Availability

The following information was supplied regarding data availability: The raw data are provided in the Supplemental File.
